# Phosphoserine-loaded chitosan membranes promote bone regeneration by activating endogenous stem cells

**DOI:** 10.3389/fbioe.2023.1096532

**Published:** 2023-03-23

**Authors:** Yue Ke, Yu Ye, Jintao Wu, Yanxia Ma, Yuxin Fang, Fei Jiang, Jinhua Yu

**Affiliations:** ^1^ Jiangsu Key Laboratory of Oral Diseases, Nanjing Medical University and Department of Endodontic, Affiliated Hospital of Stomatology, Nanjing Medical University, Nanjing, China; ^2^ Department of Stomatology, Nanjing Medical University, Nanjing, China; ^3^ Department of Periodontology, Nanjing Medical University, Nanjing, China; ^4^ Jiangsu Province Engineering Research Center of Stomatological Translational Medicine, Nanjing, China; ^5^ Department of General Dentistry, Nanjing Medical University, Nanjing, China

**Keywords:** bone regeneration, chitosan, phosphoserine, membranes, bone marrow mesenchymal stem cells

## Abstract

Bone defects that result from trauma, infection, surgery, or congenital malformation can severely affect the quality of life. To address this clinical problem, a phosphoserine-loaded chitosan membrane that consists of chitosan membranes serving as the scaffold support to accommodate endogenous stem cells and phosphoserine is synthesized. The introduction of phosphoserine greatly improves the osteogenic effect of the chitosan membranes *via* mutual crosslinking using a crosslinker (EDC, 1-ethyl-3-(3-dimethyl aminopropyl)-carbodiimide). The morphology of PS-CS membranes was shown by scanning electron microscopy (SEM) to have an interconnected porous structure. The incorporation of phosphoserine into chitosan membranes was confirmed by energy dispersive spectrum (EDS), Fourier Transforms Infrared (FTIR), and X-ray diffraction (XRD) spectrum. The CCK8 assay and Live/Dead staining, Hemolysis analysis, and cell adhesion assay demonstrated that PS-CS membranes had good biocompatibility. The osteogenesis-related gene expression of BMSCs was higher in PS-CS membranes than in CS membranes, which was verified by alkaline phosphatase (ALP) activity, immunofluorescence staining, and real-time quantitative PCR (RT-qPCR). Furthermore, micro-CT and histological analysis of rat cranial bone defect demonstrated that PS-CS membranes dramatically stimulated bone regeneration *in vivo*. Moreover, H&E staining of the main organs (heart, liver, spleen, lung, or kidney) showed no obvious histological abnormalities, revealing that PS-CS membranes were no additional systemic toxicity *in vivo*. Collectively, PS-CS membranes may be a promising candidate for bone tissue engineering.

## 1 Introduction

Bone losses resulting from trauma, infection, surgery, or congenital malformation profoundly impact the life quality of patients and remain a major clinical problem (Hao et al., 2022). Autogenous and allogenic bone grafts are common first-line treatments for bone defects, yet their use has obvious drawbacks such as donor-site availability, donor-site morbidity, and ethical concerns concerning allografts (Yao et al., 2017; Lopes et al., 2018; McNeill et al., 2020). While alternative graft materials have been used in the operation, the major disadvantages of these materials are poor osteoinductivity, unpredictable clinical outcomes, and expensive (Qian et al., 2019; Fang et al., 2020; Liang et al., 2020). Thus, the options are narrowed down to novel techniques such as tissue engineering that promises the regeneration of native-like tissues and organs (Ren et al., 2022).

Chitosan, derived from chitin, is a natural amino polysaccharide that exhibits outstanding biocompatibility, biodegradability, the capacity to form different osteogenic biomaterials, and the chelating of metal ions (Muzzarelli, 2009; Chen et al., 2013). Chitosan-based membranes have porous microstructures suitable for stem cells to migrate, adhere, and proliferate due to their chemical structure similarity to the backbone of glycosaminoglycan (GAG), the essential constituent of the extracellular matrix of bones (de Sousa Victor et al., 2020; Islam et al., 2020). Nevertheless, chitosan-based membrane itself is not osteoinductive, making it incapable of regulating osteogenic differentiation (Zou et al., 2021). Hence, bioactive components, such as ions, drugs, proteins, or growth factors (GFs), were introduced to endow chitosan membranes with better bone regeneration effects (Levengood and Zhang, 2014; Zou et al., 2021). Phosphoserine (PS) is a protein class with abundant phosphorylated amino acid residues, which can catalyze the formation of apatite crystals (Kim et al., 2016). Phosphoserine might be able to mimic the activities of a non-collagen protein such as osteopontin (OPN) (A. Reinstorf* et al., 2004; Salgado et al., 2019). OPN is a pleiotropic extracellular signal-regulated bone sialoprotein and can impact cell survival ability and migration (Paloian et al., 2016; Kadriu et al., 2018). Studies showed that OPN increased the expression of integrin β1 in BMSCs resulting in BMSC migration (Fu et al., 2019). Yang et al. (2020) proved that PS-modified biomaterials could accelerate osteogenic differentiation of MSCs by inhibiting the expression of C-X-C motif chemokine ligand 9 (Cxcl9) and increasing vascular endothelial growth factor (VEGF) secretion from osteoblasts. Cxcl9 is negative modulator of angiogenesis and osteogenesis that was proved to suppress the osteogenic differentiation of BMSCs. The molecular mechanism of Cxcl9 activation in BMSCs involved mTOR/STAT1 signaling pathway (Huang et al., 2016; Shen et al., 2019). In addition, chitosan can irreversibly bind to phosphoserine by crosslinking agents due to their special molecular structures (Kim et al., 2016; Salgado et al., 2019). Due to bright clinical applied prospects, great biocompatibility, and low prices, our groups have fabricated phosphoserine-loaded chitosan membranes and explored their bone reparation capability.

To develop a novel bone substitution graft for bone tissue engineering, we have used the freeze-drying method to synthesize phosphoserine-loaded chitosan membranes. The surface morphology and regular pore structure of the synthesized phosphoserine-loaded chitosan membranes were observed by scanning electron microscopies. Besides, the crystalline structures and elemental distributions of phosphoserine-loaded chitosan membranes were analyzed by X-ray diffraction (XRD) spectrum, energy dispersive spectrum (EDS), and Fourier Transforms Infrared (FTIR). *In vitro*, our studies proved that the phosphoserine-loaded chitosan membranes possessed good biocompatibility and stability. We further demonstrated that *in vitro* the phosphoserine-loaded chitosan membranes more efficiently promote the osteogenesis-related gene expression of BMSCs. Additionally, *in vivo* cranial defect models of phosphoserine-loaded chitosan membranes have shown significant bone regeneration as compared to chitosan membranes and untreated groups. H&E staining of the major organs were no obvious histological abnormalities, revealing that the membranes had no additional systemic toxicity *in vivo*. It showed that PS-CS membranes could modulate a better regrowth of bone, which implied they were a superior candidate for bone tissue engineering.

## 2 Materials and methods

### 2.1 Synthesis of phosphoserine-loaded chitosan membranes

The membranes were prepared by the freeze-drying process. Briefly, 2% (w/v) of chitosan (low weight molecular, Mv = 100,000) in 2% (v/v) acetic acid was freshly prepared by mixing at room temperature for 1 h. 1-ethyl-3-(3-dimethyl aminopropyl)-carbodiimide (EDC) solution was prepared as 85 mg/mL. Then 1 mL of EDC solution was reacted with 0.01 g O-phospho-L-serine (phosphoserine) for 1 h and mixed with 2% (w/v) CS for 24 h, dialyzed with 50 kDa tubes for 24 h. After that, the mixture was poured onto 12 welled culture plates and stored at −80°C. Next, the frozen samples were lyophilized at −40°C for 12 h, neutralized by immersion in a 1 M NaOH solution, and then washed with double-distilled water. The washed membranes were then stored at −40°C overnight and lyophilized to obtain the phosphoserine-chitosan membranes. 1% (w/v) of chitosan in 1% (v/v) acetic acid was freshly prepared by mixing at room temperature for 1 h. The mixture was poured onto 12 welled culture plates and stored at −80°C. Next, the frozen samples were lyophilized at −40C for 12 h, neutralized by immersion in a 1 M NaOH solution, and then washed with double-distilled water. The washed membranes were then stored at −40°C overnight and lyophilized to obtain the chitosan membranes.

### 2.2 Physical and chemical characterization of PS-CS membranes

#### 2.2.1 SEM

A scanning electron microscope (SEM, TESCAN, MAIA3, Czech Republic) was used to observe the morphology and porous of the membrane. Samples were coated with gold and were analyzed at 10 kV.

#### 2.2.2 EDS

Energy dispersive X-ray spectroscopy (EDX, TESCAN, MAIA3, Czech Republic) was used to investigate the elements’ existence and distribution on the membrane’s surface.

#### 2.2.3 Fourier transforms infrared (FT-IR) spectroscopic analysis

The chemical structures of membranes were tested by attenuated total reflection-Fourier transform infrared spectroscopy (FTIR, Thermo Scientific Nicolet iS10, United States). These membranes were lyophilized and placed on a diamond ATR window. Absorbance wavelength spectra were acquired from 4,000 to 400 cm^−1^.

#### 2.2.4 XRD

The X-ray powder diffraction (XRD, Bruker AXS, German) method was utilized to investigate the crystalline structure, its properties like crystalline phase and size, and to ensure the commonly used phase formation method. The XRD of prepared CS membranes and PS-CS membranes was measured using Cu Kα radiation (*λ* = 1.5418 Å) at room temperature, operating at a voltage of 40 kV. The experiments were performed at a scan rate of 1 min^−1^ for a scan range of 2θ = 2°–70°.

#### 2.2.5 TGA

Thermogravimetric analysis (TGA, Netzsch, Germany) was observed and captured under an argon atmosphere (60 mL/min), and samples with a definite amount were heated from 0°C to 700°C at a heating rate of 10°C min^−1^. Our study calculated weight loss (%) by considering residual weight at 700°C.

#### 2.2.6 Degradation test

To evaluate the degradation rate of the membranes, different membranes were immersed at 37°C in a cell growth medium (α-MEM, 10% FBS, and 1% P/S). At different time points, the membranes were taken out and lyophilized for weight measurement.

#### 2.2.7 Mechanical tensile testing

The tensile strengths of the CS and PS/CS were measured by a universal testing machine. The strip-shaped samples (30 × 6 × 1 mm^3^) were measured at a 5 mm/min stretching speed until rupture.

### 2.3 Isolation, cultivation, and characterization of bone marrow mesenchymal stem cells (BMSCs)

#### 2.3.1 Purification and growth of BMSCs

BMSCs were obtained from two-week-old male Sprague-Dawley rats purchased from the Animal Research Center of Nanjing Medical University. Femurs and tibias were excised aseptically. The soft tissue was removed using forceps and put clean bones in Alpha minimum essential medium (Gibco, United States). The ends of the tibia and femur were cut, and a sterile needle was inserted into the bone marrow cavity (Wu et al., 2020). The marrow cell suspension was harvested using *a*-MEM. Cells were plated in 60 mm culture dishes and incubated in *a*- MEM (Gibco, United States) with 10% fetal bovine serum (Gibco, United States), 100 U/mL penicillin (Gibco, United States), and 100 mg/mL streptomycin in a CO_2_ incubator (37°C, 5% CO_2_). The cell culture medium was replenished every 2 days. Cell passaging was performed by digestion once they reached 80%–85% confluence. Passages two through four were utilized for our study.

#### 2.3.2 Characterization of BMSCs

The BMSC phenotype was determined by labelling with fluorescent-conjugated monoclonal antibodies (BD Pharmingen, United States). The cells were collected with 0.25% trypsin-EDTA-free solution when they reached 80%. Fluorescent-conjugated antibodies (CD29, CD90, CD11a, CD45) were added and incubated under dark conditions for 1 h. Stained cells were detected using a flow cytometer after being rinsed with PBS.

#### 2.3.3 Adipogenic differentiation

BMSCs were cultured in 60 mm culture dishes until 60%–70% confluency and further incubated in an adipogenic induction medium (Cyagen Biosciences Inc, China). Cells were maintained in a CO_2_ incubator (37°C, 5% CO_2_), and the adipogenic differentiation medium was changed every 3 days. After 28 days of culture, cells were fixed and stained with Oil Red O (Ye et al., 2021).

#### 2.3.4 Chondrogenic differentiation

BMSCs (5 × 10^5^ cells) were collected and cultured in 15 mL centrifuge tubes with a chondrogenic induction medium (Cyagen Biosciences Inc, China) in a CO_2_ incubator (37°C, 5% CO_2_), and loosen the tube lids slightly. After 24 h, cell pellets were formed and changed chondrogenic induction medium every 2 days. After 28 days of culture, cell pellets were stained with Alcian Blue after fixation and frozen sections (Li et al., 2020).

### 2.4 *In vitro* studies

#### 2.4.1 Live/dead cell viability assay

The Live/Dead staining kit (Invitrogen, Carlsbad, United States) was performed on membranes cultured with BMSCs. After incubation for 1, 3, and 7 days, the samples were stained and incubated in the dark for 15 min. Upon incubation, membranes were washed with PBS (Gibco, United States) and detected by a confocal fluorescence microscope.

#### 2.4.2 CCK-8 assay

Before planting cells, CS and CS-PS samples with 6 mm diameter were sterilized by ultraviolet irradiation for 24 h. These membranes were cultured with BMSCs at a density of 2 × 10^4^ cells. After incubation for 0, 1, 3, 5, 7, and 9 days, the media was discarded, followed by the addition of 100 µL *a*- MEM medium including 10 µL Cell Counting Kit-8 reagent (Dojindo, Japan) and incubated at 37°C for 2 h. Absorbance was read at 405 nm with a microplate reader (Spectramax190, United States).

#### 2.4.3 Hemolysis assays

Fresh blood was collected from Sprague Dawley (SD) rats, and red blood cells (RBCs) were separated by centrifugation (10,000 rpm, 5 min) and washed five times with PBS. The washed RBCs were diluted and added to CS membranes or PS-CS membranes. After incubation at room temperature for 4 h, the mixed solutions were collected and centrifuged for 5 min at 4°C. Next, 100 µL supernatant of the different solutions was added to a new 96-well plate. Absorbance was read at 492 nm and calculated the hemolysis rate. Distilled water served as a positive control group, PBS as a negative control group. Hemolysis rate (%) = [(At-An)/(Ap-An)] × 100%. (At: absorbance of the test group, Ap: Absorbance of the positive control group, An: absorbance of the negative control group) (Xiao et al., 2021).

#### 2.4.4 Cell attachment

BMSCs were inoculated on different membranes at a density of 1 × 10^4^ cells. After incubation for 1 and 7 days, samples were fixed at 4°C. Then, samples were treated with Triton X-100 solution (Beyotime, China) for 10 min and stained with rhodamine phalloidin in the dark. Cell nuclei were stained with 4′, 6-Diamidino-2-phenylindole (DAPI).

#### 2.4.5 ALP activity and staining

The experiment was performed after osteogenic induction for 7 and 14 days of cultured BMSCs within membranes. ALP staining was processed using an ALP staining kit (Beyotime, China). Quantification of ALP activity was determined with an ALP activity assay kit (Jiancheng, China). Absorbance was read at 520 nm.

#### 2.4.6 Immunofluorescent staining

After being cultured in the osteogenic medium for 4, 7, and 14 days, the cells were fixed with 4% paraformaldehyde for 30 min. Then, the samples were treated with Triton X-100 solution for 5 min and blocked with normal goat serum (DCS/BioGenex, Germany) at 4°C. Specimens were treated with primary antibodies against alkaline phosphatase (ALP), runt-related transcription factor 2 (RUNX2), and osteocalcin (OCN) (R&D, Minneapolis, MN) at 4°C overnight and stained with secondary antibodies in the dark for 1 h. Finally, cell nuclei were counterstained with DAPI (Beyotime, China), which was then visualized under a laser confocal microscope.

#### 2.4.7 RT-qPCR (real-time quantitative PCR)

The osteogenic-related genes, including alkaline phosphatase (*Alp*), runt-related transcription factor 2 (*Runx2*), and osteocalcin (*Ocn*), were further investigated by RT-qPCR. The BMSCs were cultured with different membranes for 4, 7, and 14 days, followed by the isolation of total RNA using the RNA simple Total Kit (TianGen, China). The RT-qPCR evaluated the expression level of *Alp*, *Runx2*, and *Ocn* by SYBR Premix Ex Taq and the light cycler 96 system (Roche, Germany). GAPDH served as the housekeeping gene. Fold changes in gene expression were calculated by the 2^−ΔΔCT^ method. The primer sequence of each gene was noted as follows (Table 1).

**TABLE 1:** Sense and antisense primers for real-time reverse transcription PCR.

Genes	Primers	Sequences (5′–3′)
*GAPDH*	Forward	AGG​TCG​GTG​TGA​ACG​GAT​TTG
	Reverse	TGT​AGA​CCA​TGT​AGT​TGA​GGT​CA
*ALP*	Forward	TGC​CCC​TGA​CTG​AAA​TTC​CTC
	Reverse	GGG​AAG​ATA​CAA​GCC​CCA​GG
*RUNX2*	Forward	CAA​CCG​AGT​CAG​TGA​GTG​CT
	Reverse	AAG​AGG​CTG​TTT​GAC​GCC​AT
*OCN*	Forward	ATT​GTG​ACG​AGC​TAG​CGG​AC
	Reverse	TCG​AGT​CCT​GGA​GAG​TAG​CC

### 2.5 *In vivo* experiments

#### 2.5.1 Experimental procedures

To determine the bone regeneration of the PS-CS membranes *in vivo*, six-week-old male Sprague Dawley (SD) rats were used to establish the cranial defect model. The SD rats were anaesthetized with pentobarbital intraperitoneally (Nembutal, 3.5 mg per 100 g), and then midline incisions were made to expose the calvarium. The circular defect was accomplished using a 5 mm trephine bit. After implanting different membranes into defects, the soft tissue and skin were closed. The rats resumed normal activity after the operation.

#### 2.5.2 Micro-computed tomography

Four weeks after surgery, the calvarium was dissected and fixed in 4% paraformaldehyde. The calvarium was scanned using a micro-CT scanner (VivaCT 80, Switzerland). The 3-D images of bone tissue were performed using Mimics software. Micro-CT was carried out to measure the percentage of the new bone volume relative to the tissue volume (BV/TV), trabecular number (Tb. N), and bone mineral density (BMD).

#### 2.5.3 Histological analysis

The dissected calvaria was fixed, decalcified, dehydrated, and embedded in paraffin. Paraffin-embedded tissues were sectioned continuously at 10 μm thickness using a hard microtome (Leica Biosystems, Germany). Paraffin sections were stained with Hematoxylin-Eosin (H&E) and Masson. Then, sections were observed under a microscope (Olympus BX51, Japan).

Major organs were also fixed and examined using H&E staining.

### 2.6 Statistical analysis

All tests were performed in triplicates unless otherwise stated. All data were reported as the mean ± SD. Results were evaluated with one-way ANOVA or Student’s t-test. Statistical significance was set as *p* < 0.05.

## 3 Results

### 3.1 Characterization of BMSCs

**FIGURE 1:**
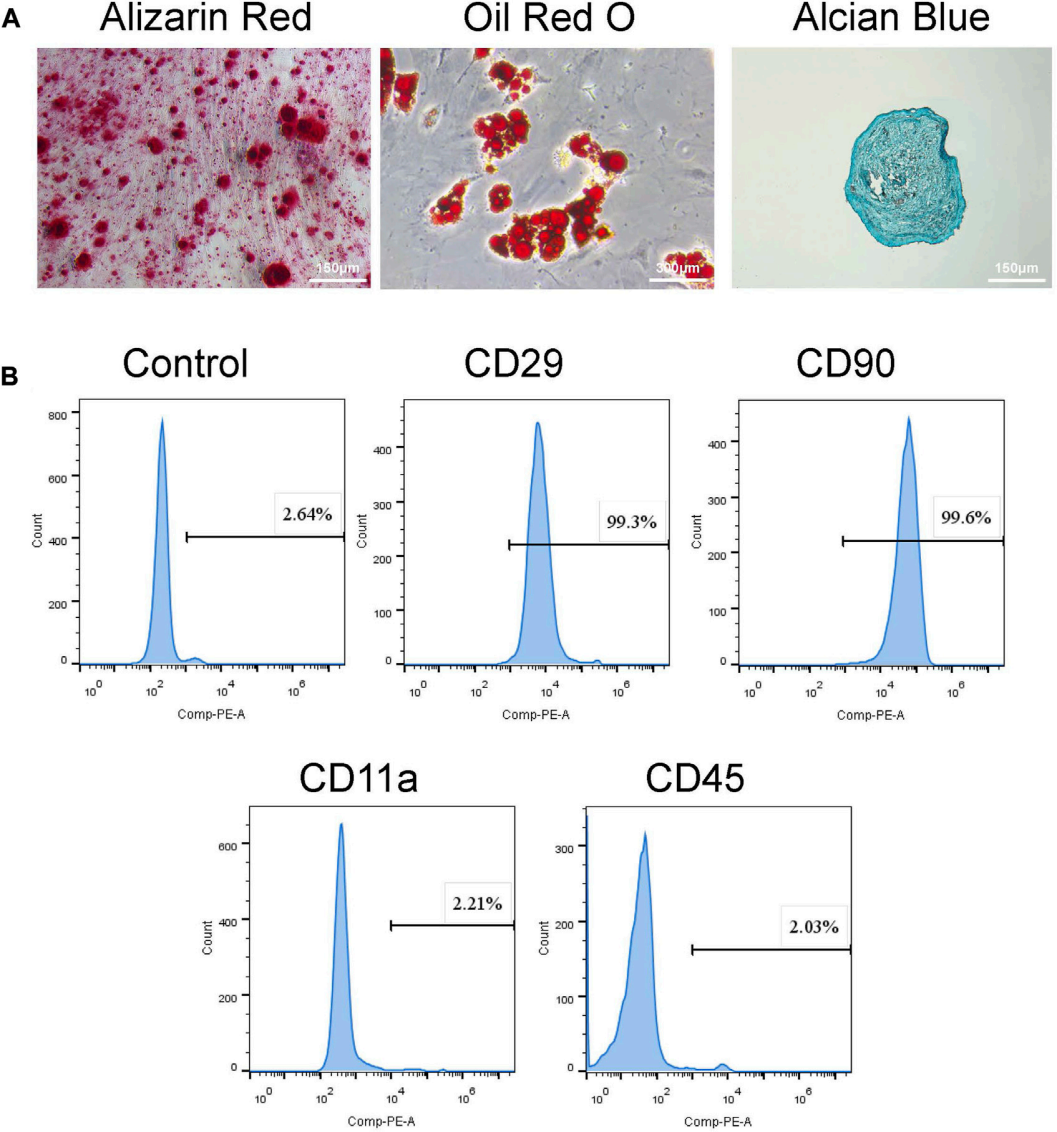
Identification of BMSCs. **(A)** Tri-lineage differentiation of BMSCs was performed *in vitro*. a. Alizarin red S staining of cells cultured for 14 days in osteogenic induction medium. b. Oil red O staining of cells cultured for 28 days in adipogenic induction medium. c. Alcian blue staining of cells cultured for 21 days in chondrogenic induction medium. **(B)** Flow cytometry demonstrated that BMSCs presented high expressions of CD29, CD90, but low expressed CD11a and CD45.

Rat BMSCs were investigated in terms of MSC cell surface epitopes. For phenotype characterization, flow cytometry analysis was performed to examine cell surface markers CD11a, CD29, CD45, and CD90 (Yang et al., 2020; He et al., 2020; Lin et al., 2020). Flow cytometry analysis showed that BMSCs positively expressed MSCs markers (CD29, CD90) while negatively expressed the hematopoietic markers such as CD11a and CD45 (Figure 1B). Moreover, Alizarin red staining and Oil Red O staining, and Alcian Blue staining confirmed osteogenic, adipogenic, and chondrogenic differentiation. Specimens were fixed and stained with Alizarin Red S, Oil Red O, and Alcian Blue (Figure 1A).

### 3.2 Physical and chemical characterization of PS-CS membranes

#### 3.2.1 Morphology of the membranes

**SCHEME 1:**
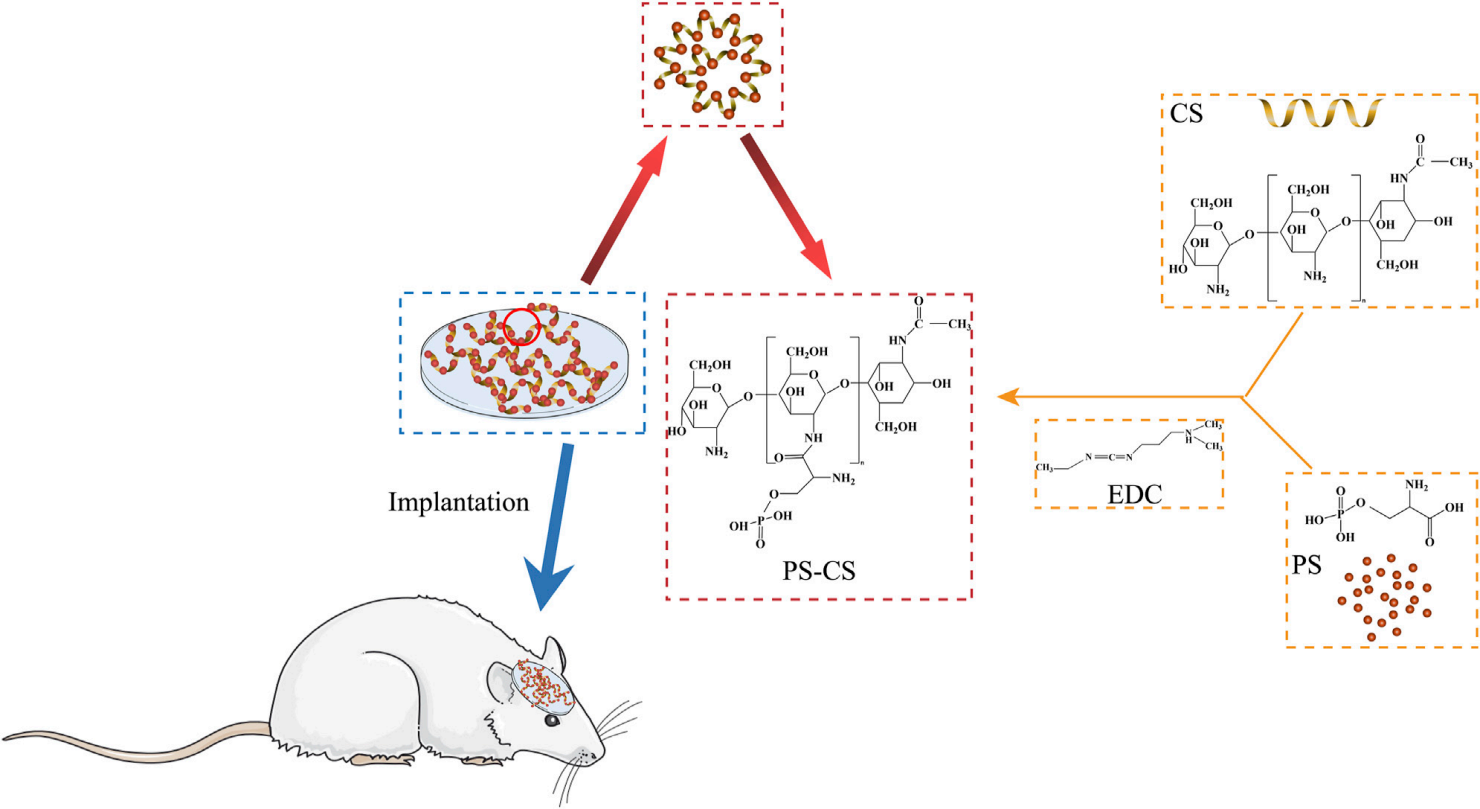
Schematic illustration for fabrication of phosphoserine-chitosan membranes.

**FIGURE 2:**
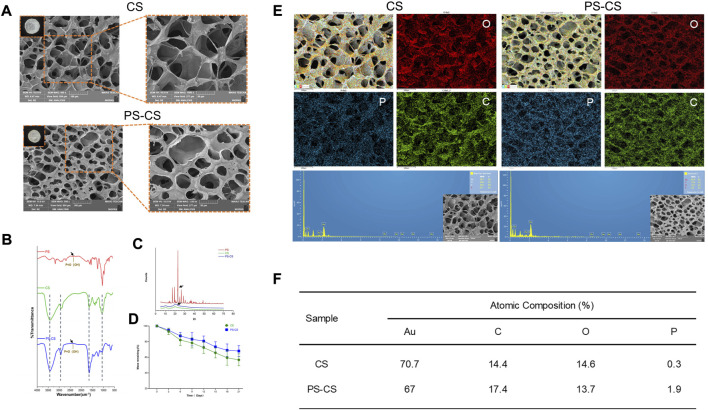
Characterization of membranes. **(A)** SEM images of fabricated membranes. **(B)** FTIR spectra of the phosphoserine powder (PS), chitosan membranes (CS), phosphoserine-chitosan membranes (PS-CS). **(C)** XRD patterns of the phosphoserine powder (PS), chitosan membranes (CS), phosphoserine-chitosan membranes (PS-CS). **(D)** The degradation profiles of chitosan membranes (CS), phosphoserine-chitosan membranes (PS-CS). **(E)** EDS elemental mapping for the merged image of all elements. **(F)** Semi-quantitative determination of elemental composition of these membranes.

The fabrication process of PS chitosan-based composite membranes is shown in Scheme 1, wherein phosphoserine was chemically crosslinked with chitosan *via* an EDC crosslinker. CS membranes were fabricated by freeze drying process alone or incorporated with phosphoserine. The SEM analysis was carried out to study the morphological characteristics of the membranes (Jindal et al., 2020). The pores present in membranes were well-formed and interconnected (Figure 2A). After being incorporated with phosphoserine, the pore distribution of PS-CS membranes was uniform. Noticeably, after crosslinking with EDC, the pore sizes of CS membranes, which can be measured according to scanning electron microscopy (SEM) images, decrease from 65 to 50 µm.

#### 3.2.2 EDS of the membranes

The elemental composition of biomaterials was investigated by energy dispersive X-ray (EDS) spectroscopy (Wu et al., 2021). Hence, EDS mapping was used to confirm that PS was incorporated in CS membranes, which showed that C, O, and P elements were in the membranes. Phosphorus (P) represents one of the most common elements in nature as well as in mammals (Masseron et al., 2020). A minimal amount of P elements may, however, mix into chitosan membranes in the process of fabricating samples. Hence the EDS mapping analysis showed that a very minute amount of P elements was distributed throughout the CS membrane. Much stronger characteristic peaks of P elements in EDS analysis confirmed the successful linkage of PS in PS-CS membranes (Figures 2E, F) compared to CS membranes.

#### 3.2.3 FTIR

The CS membranes and PS-CS membranes were investigated by FTIR (Figure 2B). CS membranes showed characteristic peaks at 3,419 cm^−1^ due to the OH and NH_2_ stretching vibrations (Shirzaei Sani et al., 2021). The peaks at 2,922 and 2,858 cm^−1^ were observed and contributed to the CH stretching. The peaks in the range of 1,155–1,093 cm^−1^ were due to bands of C-O-C linkage. The characteristic absorption bands of the phosphoserine are at 2,358cm^-1^. The FTIR spectra of PS-CS displayed characteristic absorption corresponding to the P=O(OH) group at 2,388 cm^−1^, indicating the successful crosslinking of phosphoserine with EDC in CS membranes.

#### 3.2.4 XRD

The XRD analysis of CS membranes and PS-CS membranes was measured (Figure 2C). The diffraction of CS membranes showed the characteristic crystalline peaks at 2θ of 10.3° and 19.9°, revealing the crystalline nature (Jindal et al., 2020). The phosphoserine showed a sharp peak at 23°. The PS-CS membranes displayed characteristic crystalline peaks at 2θ of 10°, 19.8°, and 22.1°.

#### 3.2.5 TGA

Thermal stability was carried out using TGA (Navidi et al., 2021). In order to probe the effects of the chemical crosslink reaction of chitosan membranes we conducted a TGA test about the membranes. The initial degradation temperature of CS membranes was similar to PS-CS membranes (Supplementary Figure S1). When the temperature reached 225°C, the thermal degradation of CS membranes was slightly higher than the PS-CS membranes. However, their complete mass loss showed the same trend because the principal component of membranes was chitosan. Obviously, presence of chemically crosslinked phosphoserine improves the thermal stability of the chitosan membranes.

#### 3.2.6 The degradation of membranes

The dry weights of different membranes were recorded for 21 days (Figure 2D). After chemical cross-linking, there was an obvious decrease in the rate of degradation of PS-CS membranes.

#### 3.2.7 Mechanical tensile testing

The influence of the phosphoserine on the mechanical property of the chitosan membrane was then investigated. The ultimate tensile strengths of the CS and PS-CS membranes were 0.91 ± 0.14 MPa and 1.15 ± 0.12 MPa, respectively (Supplementary Figure S2). It was clear that the breakdown strength and Young’s modulus were increased when 1% phosphoserine was added to the chitosan membrane.

### 3.3 *In vitro* evaluation of PS-CS membranes

#### 3.3.1 Cell proliferation

**FIGURE 3:**
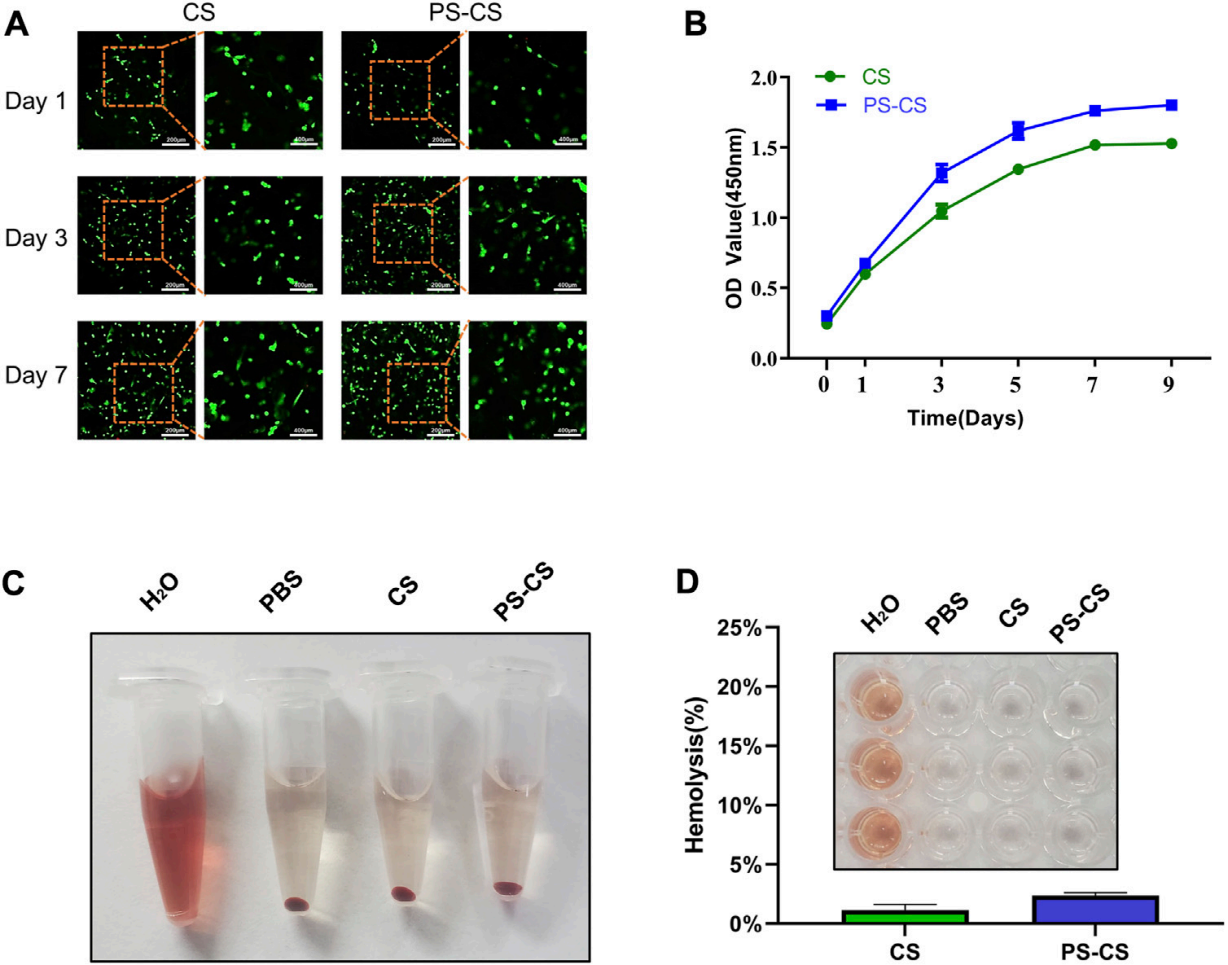
The cell viability of BMSCs in the membranes. **(A)** Live/Dead assay images of BMSC-encapsulated CS and PS-CS membranes upon 1, 3, 7 days of incubation. **(B)** CCK-8 assay of BMSC-encapsulated CS and PS-CS membranes upon 1, 3, 5, 7, 9 days of incubation. Hemolysis assays. **(C)** Images of tubes showing hemolytic activity by CS membranes and PS-CS membranes. Sterile H_2_O was used in the positive control group; PBS was used in the negative control group. **(D)** The hemolysis rate of each sample. Images of 96-well plate showing hemolytic activity by different groups of H_2_O, PBS, CS, PS-CS.

The cell proliferation of BMSCs in membranes was investigated using a Live/Dead assay (Zheng et al., 2019). The Live/Dead assay was widely used to observe cell viability and morphology. Live/Dead assay was used at one, three, and 7 days of cultured cells in CS membranes and PS-CS membranes. Staining at different time points (Figure 3A), the survival of BMSCs attained excellent viability and a high cell population, which indicated that the membranes were not cytotoxic and had outstanding biocompatibility. On days 3 and 7, the PS-CS had more live cells than the CS. The integration of PS yielded significant positive effects. It was further demonstrated by CCK-8 that BMSCs grown on the PS-CS had significantly higher viability than on the CS (Figure 3B).

#### 3.3.2 Hemolysis assays

Hemolysis assays were used to test the biocompatibility of CS and PS-CS with blood cells (Xiao et al., 2021). Hemolysis analysis showed no obvious difference in hemolysis among rat RBCs incubated at CS membranes and PS-CS membranes (Figures 3C, D).

#### 3.3.3 Cell adhesion

**FIGURE 4:**
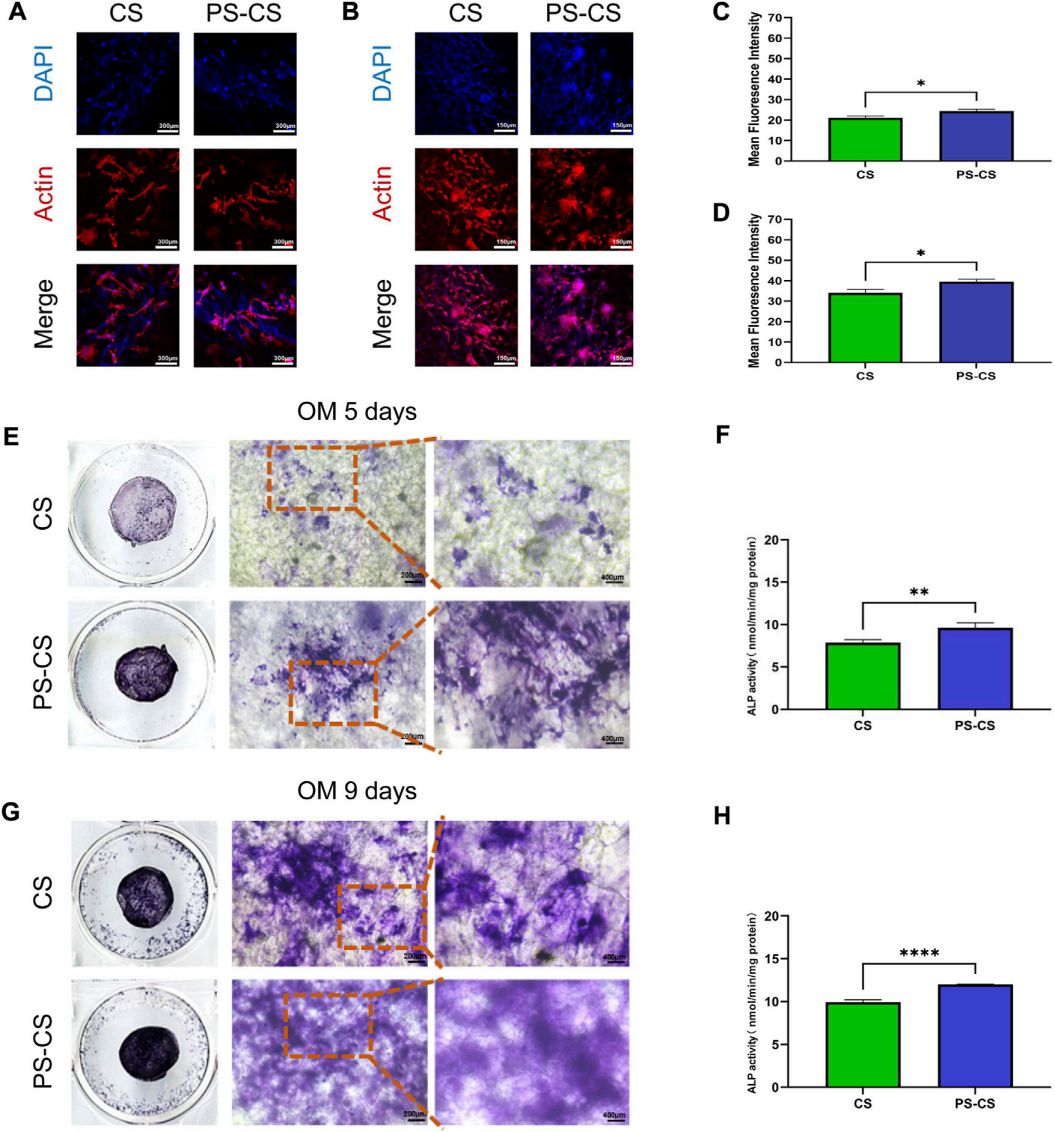
Cell attachment assay. **(A, B)** Fluorescent staining of cultured cells over the membrane after culturing for 24 h **(A)**, 7 days **(B)**. Red, cytoskeleton stained by rhodamine phalloidin; Blue, nuclei stained by DAPI. **(C, D)** Quantitative analysis of the fluorescent intensity after cell attachment on the scaffolds at 24 h **(C)**, and 7 days **(D)** post seeding. Effect of CS membranes (CS) and phosphoserine-chitosan membranes (PS-CS) on ALP activity and mineralization in BMSCs. **(E, G)** ALP staining of BMSCs after cultured with membranes for 5 days **(E)**, 9 days **(G)**. **(F, H)** ALP activities of BMSCs cultured on different membranes for 5 days **(F)** and 9 days **(H)**. The data were analyzed statistically using analysis of variance (ANOVA). *p < 0.05; **p < 0.01; and ***p < 0.001, ****p < 0.0001.

BMSCs’ morphology and adhesion were visualized using confocal microscopy (Yu et al., 2023). Staining on days 1 and 7 (Figures 4A, B), the BMSCs randomly adhered to membranes, and their filopodia were extended, indicating ideal biocompatibility for all groups. However, the fluorescence intensities of the PS-CS membranes were stronger than the CS membranes, demonstrating that PS-CS membranes were more suitable for BMSCs to migrate, adhere to, and proliferate (Figures 4C, D).

#### 3.3.4 ALP staining and activity measurement

Differentiation of BMSCs to osteoblasts can be evaluated by ALP activity (Li et al., 2020; Oh et al., 2021). ALP is a membrane-bound enzyme responsible for the extracellular matrix’s mineralization. The osteogenic effects of the PS-CS membranes were tested by ALP staining and ALP activity measurements. After 5 days of culture, the ALP staining of the PS-CS membranes was deeper than the CS membranes (Figure 4E). On day 9 (Figure 4G), the ALP staining of the PS-CS membranes was higher than the CS membranes. Moreover, ALP activity levels on days 5 and 9 (Figures 4F, H) revealed the same tendency with the ALP staining. These results indicated that BMSCs grown on PS-CS membranes could achieve higher ALP expression levels.

#### 3.3.5 Immunofluorescence staining

**FIGURE 5:**
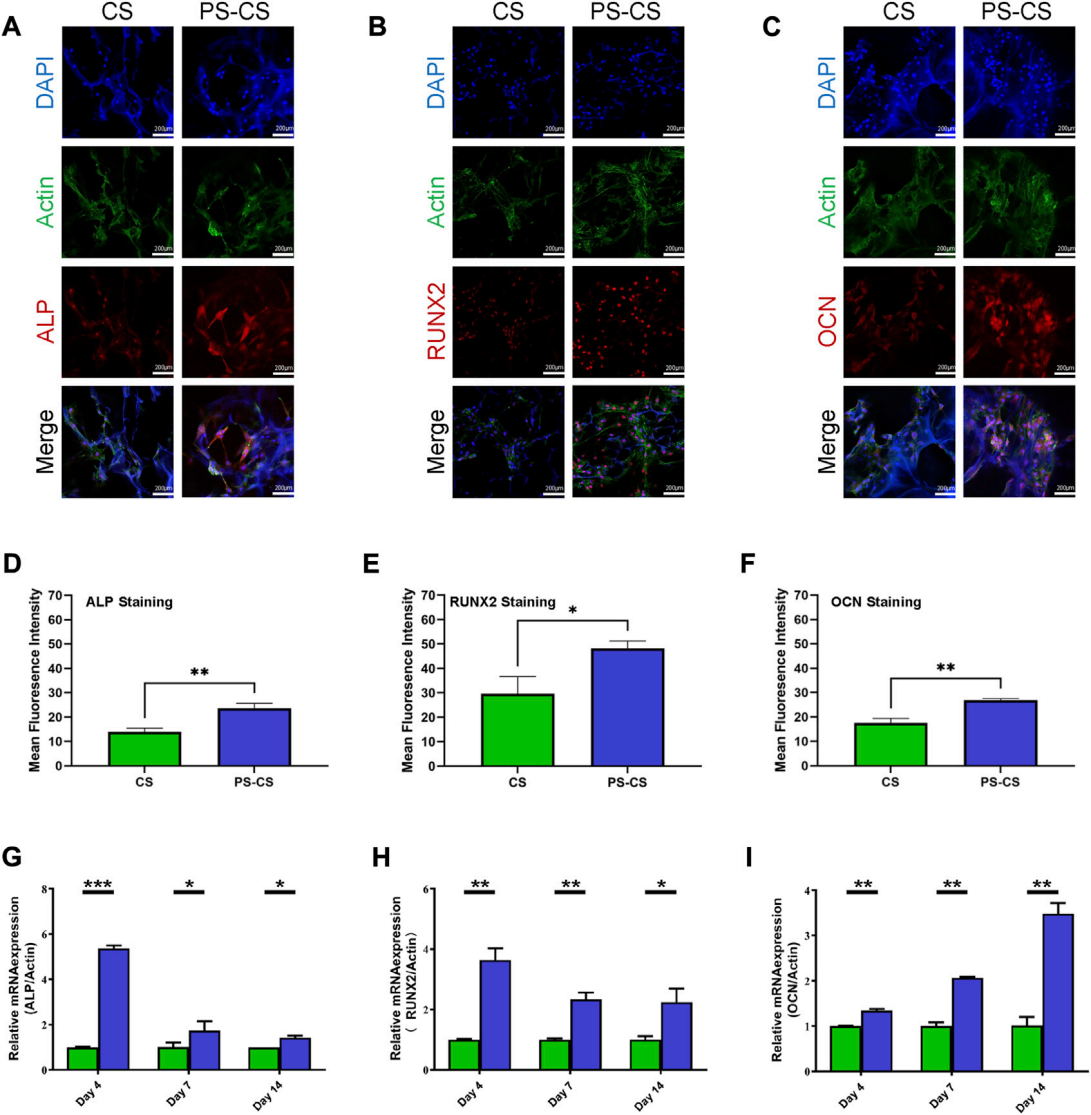
Immunofluorescent staining of osteogenic-related markers. **(A)**: ALP staining of BMSCs cultured on the membranes for 5 days. **(B)**: RUNX2 staining of BMSCs cultured on the membranes for 9 days. **(C)**: OCN staining of BMSCs cultured on the membranes for 14 days. **(D–F)**: Quantitative analysis of the fluorescent intensity of the ALP **(D)**, RUNX2 **(E)**, and OCN **(F)**. Expression of osteogenic-related genes in BMSCs. **(G–I)** qRT-PCR evaluation of osteogenic gene ALP **(G)**, RUNX2 **(H)**, OCN **(I)**. The data were analyzed statistically using analysis of variance (ANOVA). *p < 0.05; **p < 0.01; and ***p < 0.001, ****p < 0.0001.

ALP and RUNX2 are typical osteogenic differentiation markers in the earlier stage. Moreover, immunofluorescent staining was carried out to investigate the osteogenic induction qualitatively. The expression of ALP was investigated (Figure 5A). The membranes were stained with antibodies against ALP (red), Actin (green), and nuclei (blue). All of them expressed ALP, but the CS membranes were relatively weak. The samples were stained with antibodies against RUNX2 (red), Actin (green), and nuclei (blue) (Figure 5B). They all expressed RUNX2, but stronger fluorescence intensities were displayed in the PS-CS membranes. OCN is a late-stage marker of osteoblast differentiation. Furthermore, the immunofluorescence staining of OCN showed the same results (Figure 5C). These results proved that the PS-CS membranes could enhance the expression of key osteogenic markers. After immunofluorescent staining, quantitative analysis was performed on each image using ImageJ (Figures 5D–F).

#### 3.3.6 Expression of osteogenic-associated genes in BMSCs

To further verify these results, we employed RT-qPCR to detect the expression of these genes. The PS-CS membranes greatly upregulated the expression of osteogenic markers *Alp* and *Runx2* compared with CS membranes on day 4. The expression level of *Ocn* was strongly promoted in PS-CS membranes on day 14. Based on these results, cells on PS-CS membranes can substantially promote osteogenic gene expression compared to the CS membranes (Figures 5G–I).

### 3.4 *In vivo* evaluation of PS-CS membranes

#### 3.4.1 Micro-CT analysis

**FIGURE 6:**
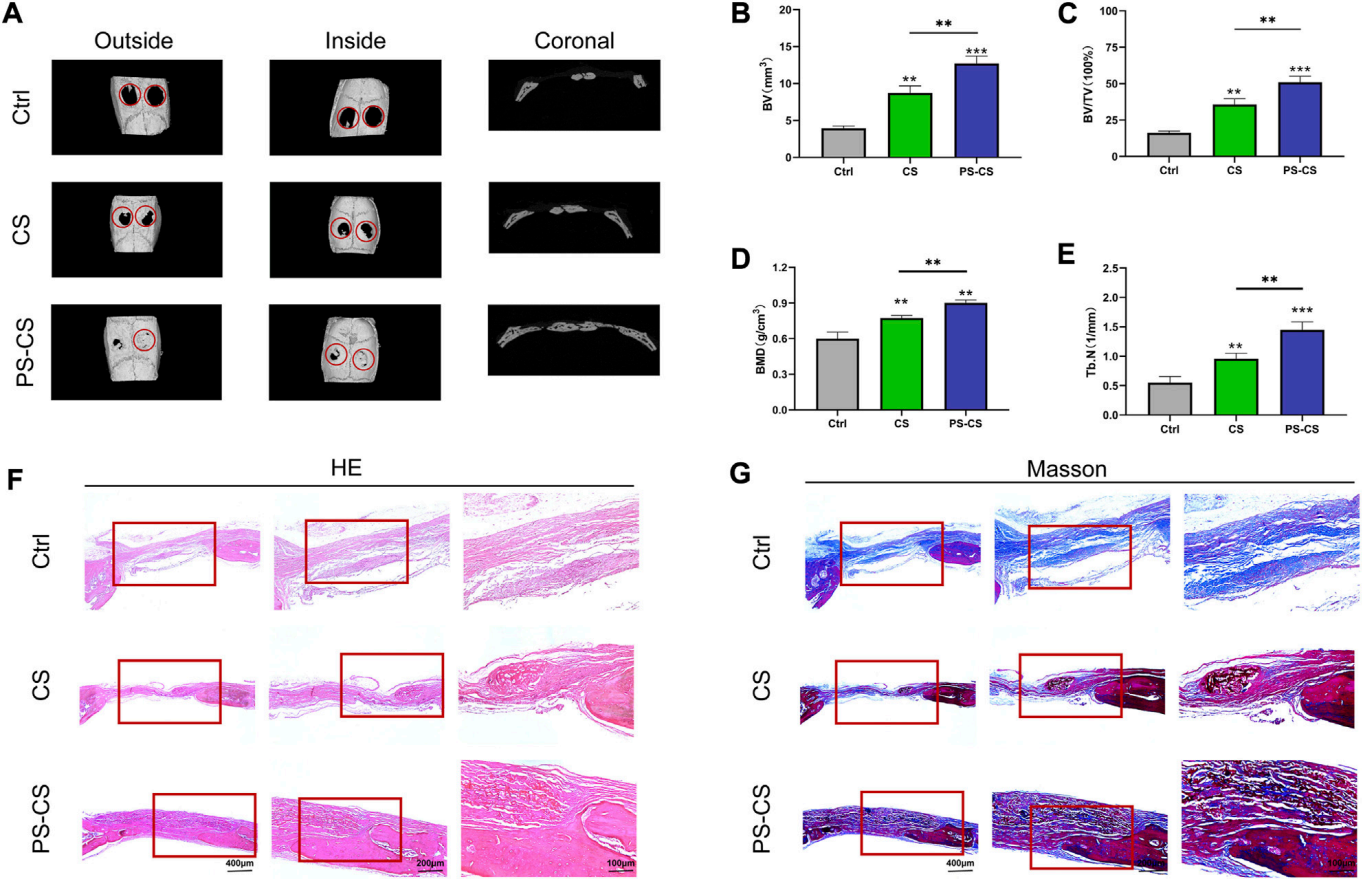
*In vivo* bone regeneration. **(A)** Micro-CT images of the bone defects in rats 4 weeks after implantation of different membranes. **(B–E)** Quantitative analysis of the bone formation in the defects, BV **(B)**, BV/TV **(C)**, BMD **(D)**, Th. N **(E)**. **(F, G)** HE and Masson’s staining of rat cranial defects after implanted with CS membranes or PS-CS membranes for 4 weeks. The data were analyzed statistically using analysis of variance (ANOVA). *p < 0.05; **p < 0.01; and ***p < 0.001, ****p < 0.0001.

The osteogenic inducing capabilities of PS-CS membranes have not been tested *in vivo*. Four weeks after surgery, all rats were sacrificed for micro-CT scanning (Xiao et al., 2021). The micro-CT scanning demonstrated limited regenerated bone in the control groups and only a small amount of newly formed bone in the CS groups. In contrast, a higher amount of bone regeneration was observed in the PS-CS groups (Figure 6A). These data were further analyzed for calculating the BV, BV/TV, BMD, and Tb. N (Figures 6B–E). Our results showed that PS-CS membranes could promote bone formation *in vivo*.

#### 3.4.2 Histological analysis

After micro-CT analysis, specimens were evaluated histologically (Han et al., 2019). As shown by H&E staining images (Figure 6F), the control group was not filled with membranes and had little bone formation, while in implant groups (CS and PS-CS), new bone restored the defect along the membranes. Substantially more bone regeneration was in the PS-CS groups than in the CS groups, reflected in much more mature new bone formation in 4 weeks. Because Masson’s trichrome staining can clearly distinguish bone and fibrous tissues, Masson staining images were locally enlarged to further investigate defect parts’ structure (Figure 6G). In the control group of 4 weeks, very limited newly formed bone filled defects. In the CS membrane group, a small amount of regeneration bone was observed surrounding the edge of membranes in the fourth week. In the PS-CS membrane group, new bone was formed along the membrane in the fourth week.

H&E staining of the heart, liver, spleen, lung, and kidney was performed to evaluate the systematic toxicity of membranes (Supplementary Figure S3) (Gao et al., 2019). There were no obvious histopathological differences in the main organs, revealing that the membranes had no additional systemic toxicity *in vivo*.

## 4 Discussion

As a common clinical problem, repairing and regenerating damaged bone tissue remain a challenge (Lee et al., 2019; Xie et al., 2021). Advances in bone tissue engineering provide new therapeutic strategies for bone defect repair, and tissue engineering strategies that use chitosan-based bone-filling materials to repair bone defects have achieved good effectiveness (LogithKumar et al., 2016; Tao et al., 2020; Yadav et al., 2021). Most types of injectable chitosan-based hydrogels lack osteoconductivity that is due to their continuous microstructure without any pores (Cui et al., 2019). However, the PS-CS membranes have highly uniform pore structures in our work, which also displayed good osteoconductivity (Figure 2A). Meanwhile, a large number of bioactive components have been used to modify bone-regenerative biomaterials, which would be expected to influence the biomaterial’s biological activity and, as a result, bone formation (Schmidt-Bleek et al., 2016; Martin and Bettencourt, 2018; Fu et al., 2021). Compared with some bioactive components, phosphoserine as a bioactive protein offered some unique advantages, including great biocompatibility, non-toxicity, ideal biodegradation, and significant osteoinductivity (Kim et al., 2016). In this work, we introduced phosphoserine to endow chitosan membranes for repairing bone defects.

Chitosan has been used in a wide variety of regenerative medicine because of its superior biological properties and low prices (Prabaharan and Sivashankari, 2016; Chen et al., 2018; Rajabi et al., 2021). Phosphoserine-loaded chitosan membrane is a chitosan-derived biomaterial, which greatly reduces the cost of manufacturing. In addition, the existence of *ß*-(1,4) glycosidic bonds between D-glucosamine and N-acetyl-D-glucosamine makes chitosan easy to be modified by chemical reactions with an ideal osteogenic capacity (Islam et al., 2020). Phosphoserine is a specialized type of functional protein that serves unique chemical bonds to achieve chemical cross-links in chitosan membranes. This property makes this chemical cross-linking membrane different from simple physical mixing in which crosslinks are made of stable covalent bonds. Covalent bonds make these membrane-like functional biomaterials far more robust under a variety of conditions, making the material widely usable.

When phosphoserine is incorporated into the membrane, this phosphorylated amino acid can catalyze the formation of apatite crystals (Kim et al., 2016). *In vitro* studies, the phosphoserine-loaded chitosan membranes have a regular pore structure, a suitable degradation rate, and a suitable microenvironment (Figure 2D; Figure 3). Those structure characteristics are extremely important in biomaterial-based tissue engineering strategies, because bone formation is a complex, highly coordinated process that involve infiltration, proliferation and osteogenic differentiation of the endogenous stem cells, bone matrix deposition, and vascular invasion. We further validated the utility of PS-CS membranes as osteogenic biomaterials by ALP staining, immunofluorescence staining, and RT-qPCR (Figures 4E–G; Figure 5). The previous reports proved that phosphoserine-functionalized branches of the dendrons could downregulate C-X-C motif chemokine ligand 9 (Cxcl9) gene expressions (Yang et al., 2020). Some scholars have confirmed that Cxcl9 could be released by osteoblasts, which suppressed osteogenesis-associated gene expressions by binding to vascular endothelial growth factor (VEGF) and inhibiting VEGF binding to endothelial cells and osteoblasts (Han et al., 2011; Duan et al., 2016; Hu and Olsen, 2016; Huang et al., 2016). However, the specific mechanisms by which phosphoserine-loaded chitosan membranes promote osteogenesis were not assessed in our study, although previous studies suggest that phosphoserine and cxcl-9 may be implicated. That is the direction we need to work on in the future.

To evaluate these effects *in vivo* we transplanted membranes into calvarial defects in rats. The satisfactory results of *in vitro* experiments were yielded in the calvarial defect model only by transplanting the material itself in the absence of cells, small molecular drugs, or growth factors (Figure 6). This feature illustrates that the PS-CS membranes can promote endogenous bone marrow mesenchymal stem cell infiltration, proliferation, and *in situ* differentiation in the absence of any growth factors, or small molecular drugs. Membrane-like functional biomaterials have been generally employed in different types of bone defects, while these are unsuited for treating large-sized bone defects due to their spatial structure (Verrier et al., 2016; Stahl and Yang, 2021). It follows for this reason that PS-CS membrane alone is unable to treat non-healing segmental bone defects. On the one hand, some previous studies proved that the structure of chitosan-based biomaterials were changed after adding hydroxyapatite and montmorillonite (MMT)-type clay (Farokhi et al., 2018; Koc Demir et al., 2018; Cui et al., 2019; Arcos and Vallet-Regi, 2020; Kaushik et al., 2020). Maybe we can integrate the superiorities of PS-CS membranes in osteoinductivity and the merits of MMT in mechanical strength to form MMT/PS-CS membranes. On the other hand, Hao et al. (2022) has shown that by combining the bone regeneration units (BRUs) with the superiorities of decalcified bone matrix (DBM) scaffolds in mechanical properties, a BRU-loaded DBM framework successfully applied to remodel large structural bone defects. Maybe we can fabricate BMSC-loaded PS-CS membranes to form BRUs, the treatment of non-healing segmental bone defects with PS-CS membrane combined with DBM scaffolds can expect.

## 5 Conclusion

To sum up, we successfully developed the PS-CS membranes and explored their function in BMSCs. The XRD, FT-IR, and EDS results confirmed that phosphoserine successfully conjugated the CS membranes. *In vitro* experiments revealed that PS-CS membranes promoted osteogenesis differentiation of BMSCs compared with CS membranes. *In vivo* experiments showed that PS-CS membranes meaningfully improved bone regeneration compared to CS membranes, which further verified the potential effects of this biomaterial in bone tissue engineering.

## Data Availability

The raw data supporting the conclusion of this article will be made available by the authors, without undue reservation.

## Supplementary material

SUPPLEMENTARY FIGURE S1 The TGA diagram of chitosan membranes (CS), phosphoserine-chitosan membranes (PS-CS).

SUPPLEMENTARY FIGURE S2 The mechanical properties of membranes. **(A)** Strain at failure, **(B)** ultimate tensile stress, **(C)** Young’s modulus (n = 3).

SUPPLEMENTARY FIGURE S3 H&E staining of the major organs (heart, liver, spleen, lung, kidney) after implanting membranes.

## References

[B1] Arcos D. Vallet-Regi M. (2020). Substituted hydroxyapatite coatings of bone implants. J. Mater Chem. B 8 (9), 1781–1800. 10.1039/c9tb02710f 32065184 PMC7116284

[B41] A. Reinstorf* M. R. Gelinsky M. Pompe W. Hempel U. Wenzel K.-W. Simon P. (2004). Phosphoserine ± a convenient compound for modi®cation of calcium phosphate bone cement collagen composites. J. Mater. Sci. Mater. Med. 15, 4. 10.1023/B:JMSM.0000021119.14870.3d 15332616

[B2] Chen C. Liu Y. Wang H. Chen G. Wu X. Ren J. (2018). Multifunctional chitosan inverse opal particles for wound healing. ACS Nano 12 (10), 10493–10500. 10.1021/acsnano.8b06237 30256608

[B3] Chen M. C. Mi F. L. Liao Z. X. Hsiao C. W. Sonaje K. Chung M. F. (2013). Recent advances in chitosan-based nanoparticles for oral delivery of macromolecules. Adv. Drug Deliv. Rev. 65 (6), 865–879. 10.1016/j.addr.2012.10.010 23159541

[B4] Cui Z. K. Kim S. Baljon J. J. Wu B. M. Aghaloo T. Lee M. (2019). Microporous methacrylated glycol chitosan-montmorillonite nanocomposite hydrogel for bone tissue engineering. Nat. Commun. 10 (1), 3523. 10.1038/s41467-019-11511-3 31388014 PMC6684526

[B5] de Sousa Victor R. Marcelo da Cunha Santos A. Viana de Sousa B. de Araujo Neves G. Navarro de Lima Santana L. Rodrigues Menezes R. (2020). A review on chitosan's uses as biomaterial: Tissue engineering, drug delivery systems and cancer treatment. Mater. (Basel) 13 (21), 4995. 10.3390/ma13214995 PMC7664280 33171898

[B6] Duan X. Bradbury S. R. Olsen B. R. Berendsen A. D. (2016). VEGF stimulates intramembranous bone formation during craniofacial skeletal development. Matrix Biol. 52-54, 127–140. 10.1016/j.matbio.2016.02.005 26899202 PMC4875795

[B7] Fang J. Liu R. Chen S. Liu Q. Cai H. Lin Y. (2020). Tuning the immune reaction to manipulate the cell-mediated degradation of a collagen barrier membrane. Acta Biomater. 109, 95–108. 10.1016/j.actbio.2020.03.038 32268238

[B8] Farokhi M. Mottaghitalab F. Samani S. Shokrgozar M. A. Kundu S. C. Reis R. L. (2018). Silk fibroin/hydroxyapatite composites for bone tissue engineering. Biotechnol. Adv. 36 (1), 68–91. 10.1016/j.biotechadv.2017.10.001 28993220

[B9] Fu J. Wang Y. Jiang Y. Du J. Xu J. Liu Y. (2021). Systemic therapy of MSCs in bone regeneration: A systematic review and meta-analysis. Stem Cell. Res. Ther. 12 (1), 377. 10.1186/s13287-021-02456-w 34215342 PMC8254211

[B10] Fu X. Liu G. Halim A. Ju Y. Luo Q. Song A. G. (2019). Mesenchymal stem cell migration and tissue repair. Cells 8 (8), 784. 10.3390/cells8080784 31357692 PMC6721499

[B11] Gao F. Li W. Deng J. Kan J. Guo T. Wang B. (2019). Recombinant human hair keratin nanoparticles accelerate dermal wound healing. ACS Appl. Mater Interfaces 11 (20), 18681–18690. 10.1021/acsami.9b01725 31038908

[B12] Han X. Wu Z. Di J. Pan Y. Zhang H. Du Y. (2011). CXCL9 attenuated chemotherapy-induced intestinal mucositis by inhibiting proliferation and reducing apoptosis. Biomed. Pharmacother. 65 (8), 547–554. 10.1016/j.biopha.2011.03.008 21775092

[B13] Han X. Zhou X. Qiu K. Feng W. Mo H. Wang M. (2019). Strontium-incorporated mineralized PLLA nanofibrous membranes for promoting bone defect repair. Colloids Surf. B Biointerfaces 179, 363–373. 10.1016/j.colsurfb.2019.04.011 30999115

[B14] Hao J. Bai B. Ci Z. Tang J. Hu G. Dai C. (2022). Large-sized bone defect repair by combining a decalcified bone matrix framework and bone regeneration units based on photo-crosslinkable osteogenic microgels. Bioact. Mater 14, 97–109. 10.1016/j.bioactmat.2021.12.013 35310359 PMC8892219

[B15] He L. He T. Xing J. Zhou Q. Fan L. Liu C. (2020). Bone marrow mesenchymal stem cell-derived exosomes protect cartilage damage and relieve knee osteoarthritis pain in a rat model of osteoarthritis. Stem Cell. Res. Ther. 11 (1), 276. 10.1186/s13287-020-01781-w 32650828 PMC7350730

[B16] Hu K. Olsen B. R. (2016). The roles of vascular endothelial growth factor in bone repair and regeneration. Bone 91, 30–38. 10.1016/j.bone.2016.06.013 27353702 PMC4996701

[B17] Huang B. Wang W. Li Q. Wang Z. Yan B. Zhang Z. (2016). Osteoblasts secrete Cxcl9 to regulate angiogenesis in bone. Nat. Commun. 7, 13885. 10.1038/ncomms13885 27966526 PMC5171795

[B18] Islam M. M. Shahruzzaman M. Biswas S. Nurus Sakib M. Rashid T. U. (2020). Chitosan based bioactive materials in tissue engineering applications-A review. Bioact. Mater 5 (1), 164–183. 10.1016/j.bioactmat.2020.01.012 32083230 PMC7016353

[B19] Jindal A. Mondal T. Bhattacharya J. (2020). An *in vitro* evaluation of zinc silicate fortified chitosan scaffolds for bone tissue engineering. Int. J. Biol. Macromol. 164, 4252–4262. 10.1016/j.ijbiomac.2020.09.018 32910962

[B20] Kadriu B. Gold P. W. Luckenbaugh D. A. Lener M. S. Ballard E. D. Niciu M. J. (2018). Acute ketamine administration corrects abnormal inflammatory bone markers in major depressive disorder. Mol. Psychiatry 23 (7), 1626–1631. 10.1038/mp.2017.109 28555075 PMC5709243

[B21] Kaushik N. Nhat Nguyen L. Kim J. H. Choi E. H. Kumar Kaushik N. (2020). Strategies for using polydopamine to induce biomineralization of hydroxyapatite on implant materials for bone tissue engineering. Int. J. Mol. Sci. 21, 6544. 10.3390/ijms21186544 32906793 PMC7555775

[B22] Kim S. Cui Z. K. Fan J. Fartash A. Aghaloo T. L. Lee M. (2016). Photocrosslinkable chitosan hydrogels functionalized with the RGD peptide and phosphoserine to enhance osteogenesis. J. Mater Chem. B 4 (31), 5289–5298. 10.1039/C6TB01154C 28044100 PMC5200955

[B23] Koc Demir A. Elcin A. E. Elcin Y. M. (2018). Strontium-modified chitosan/montmorillonite composites as bone tissue engineering scaffold. Mater Sci. Eng. C Mater Biol. Appl. 89, 8–14. 10.1016/j.msec.2018.03.021 29752122

[B24] Lee J. Byun H. Madhurakkat Perikamana S. K. Lee S. Shin H. (2019). Current advances in immunomodulatory biomaterials for bone regeneration. Adv. Healthc. Mater 8 (4), e1801106. 10.1002/adhm.201801106 30328293

[B25] Levengood S. L. Zhang M. (2014). Chitosan-based scaffolds for bone tissue engineering. J. Mater Chem. B 2 (21), 3161–3184. 10.1039/C4TB00027G 24999429 PMC4078888

[B26] Li Y. Bian M. Zhou Z. Wu X. Ge X. Xiao T. (2020). Circular RNA SIPA1L1 regulates osteoblastic differentiation of stem cells from apical papilla via miR-204-5p/ALPL pathway. Stem Cell. Res. Ther. 11 (1), 461. 10.1186/s13287-020-01970-7 33138854 PMC7607702

[B27] Liang Y. Luan X. Liu X. (2020). Recent advances in periodontal regeneration: A biomaterial perspective. Bioact. Mater 5 (2), 297–308. 10.1016/j.bioactmat.2020.02.012 32154444 PMC7052441

[B28] Lin M. Liu X. Zheng H. Huang X. Wu Y. Huang A. (2020). IGF-1 enhances BMSC viability, migration, and anti-apoptosis in myocardial infarction via secreted frizzled-related protein 2 pathway. Stem Cell. Res. Ther. 11 (1), 22. 10.1186/s13287-019-1544-y 31918758 PMC6953226

[B29] LogithKumar R. KeshavNarayan A. Dhivya S. Chawla A. Saravanan S. Selvamurugan N. (2016). A review of chitosan and its derivatives in bone tissue engineering. Carbohydr. Polym. 151, 172–188. 10.1016/j.carbpol.2016.05.049 27474556

[B30] Lopes D. Martins-Cruz C. Oliveira M. B. Mano J. F. (2018). Bone physiology as inspiration for tissue regenerative therapies. Biomaterials 185, 240–275. 10.1016/j.biomaterials.2018.09.028 30261426 PMC6445367

[B31] Martin V. Bettencourt A. (2018). Bone regeneration: Biomaterials as local delivery systems with improved osteoinductive properties. Mater Sci. Eng. C Mater Biol. Appl. 82, 363–371. 10.1016/j.msec.2017.04.038 29025670

[B32] Masseron T. Garcia-Hernandez D. A. Santovena R. Manchado A. Zamora O. Manteiga M. (2020). Phosphorus-rich stars with unusual abundances are challenging theoretical predictions. Nat. Commun. 11 (1), 3759. 10.1038/s41467-020-17649-9 32753582 PMC7403594

[B33] McNeill E. P. Zeitouni S. Pan S. Haskell A. Cesarek M. Tahan D. (2020). Characterization of a pluripotent stem cell-derived matrix with powerful osteoregenerative capabilities. Nat. Commun. 11 (1), 3025. 10.1038/s41467-020-16646-2 32541821 PMC7295745

[B34] Muzzarelli R. A. A. (2009). Chitins and chitosans for the repair of wounded skin, nerve, cartilage and bone. Carbohydr. Polym. 76 (2), 167–182. 10.1016/j.carbpol.2008.11.002

[B35] Navidi G. Allahvirdinesbat M. Al-Molki S. M. M. Davaran S. Panahi P. N. Aghazadeh M. (2021). Design and fabrication of M-SAPO-34/chitosan scaffolds and evaluation of their effects on dental tissue engineering. Int. J. Biol. Macromol. 187, 281–295. 10.1016/j.ijbiomac.2021.07.104 34314794

[B36] Oh Y. Ahn C. B. Marasinghe M. Je J. Y. (2021). Insertion of gallic acid onto chitosan promotes the differentiation of osteoblasts from murine bone marrow-derived mesenchymal stem cells. Int. J. Biol. Macromol. 183, 1410–1418. 10.1016/j.ijbiomac.2021.05.122 34022306

[B37] Paloian N. J. Leaf E. M. Giachelli C. M. (2016). Osteopontin protects against high phosphate-induced nephrocalcinosis and vascular calcification. Kidney Int. 89 (5), 1027–1036. 10.1016/j.kint.2015.12.046 27083280 PMC4834144

[B38] Prabaharan M. Sivashankari P. S. (2016). “Chitin and Chitosan for Regenerative Medicine,” in Springer Series on Polymer and Composite Materials. Editors Dutta P. K. (New Delhi Heidelberg New York Dordrecht London: Springer). 10.1007/978-81-322-2511-9

[B39] Qian Y. Zhou X. Zhang F. Diekwisch T. G. H. Luan X. Yang J. (2019). Triple PLGA/PCL scaffold modification including silver impregnation, collagen coating, and electrospinning significantly improve biocompatibility, antimicrobial, and osteogenic properties for orofacial tissue regeneration. ACS Appl. Mater Interfaces 11 (41), 37381–37396. 10.1021/acsami.9b07053 31517483 PMC7220812

[B40] Rajabi M. McConnell M. Cabral J. Ali M. A. (2021). Chitosan hydrogels in 3D printing for biomedical applications. Carbohydr. Polym. 260, 117768. 10.1016/j.carbpol.2021.117768 33712126

[B42] Ren S. Zhou Y. Zheng K. Xu X. Yang J. Wang X. (2022). Cerium oxide nanoparticles loaded nanofibrous membranes promote bone regeneration for periodontal tissue engineering. Bioact. Mater 7, 242–253. 10.1016/j.bioactmat.2021.05.037 34466730 PMC8379477

[B43] Salgado C. L. Teixeira B. I. B. Monteiro F. J. M. (2019). Biomimetic composite scaffold with phosphoserine signaling for bone tissue engineering application. Front. Bioeng. Biotechnol. 7, 206. 10.3389/fbioe.2019.00206 31552233 PMC6743420

[B44] Schmidt-Bleek K. Willie B. M. Schwabe P. Seemann P. Duda G. N. (2016). BMPs in bone regeneration: Less is more effective, a paradigm-shift. Cytokine Growth Factor Rev. 27, 141–148. 10.1016/j.cytogfr.2015.11.006 26678813

[B45] Shen Q. Fan X. Jiang M. Ye Z. Zhou Y. Tan W. S. (2019). Inhibiting expression of Cxcl9 promotes angiogenesis in MSCs-HUVECs co-culture. Arch. Biochem. Biophys. 675, 108108. 10.1016/j.abb.2019.108108 31550444

[B46] Shirzaei Sani I. Rezaei M. Baradar Khoshfetrat A. Razzaghi D. (2021). Preparation and characterization of polycaprolactone/chitosan-g-polycaprolactone/hydroxyapatite electrospun nanocomposite scaffolds for bone tissue engineering. Int. J. Biol. Macromol. 182, 1638–1649. 10.1016/j.ijbiomac.2021.05.163 34052267

[B47] Stahl A. Yang Y. P. (2021). Regenerative approaches for the treatment of large bone defects. Tissue Eng. Part B Rev. 27 (6), 539–547. 10.1089/ten.TEB.2020.0281 33138705 PMC8739850

[B48] Tao F. Cheng Y. Shi X. Zheng H. Du Y. Xiang W. (2020). Applications of chitin and chitosan nanofibers in bone regenerative engineering. Carbohydr. Polym. 230, 115658. 10.1016/j.carbpol.2019.115658 31887899

[B49] Verrier S. Alini M. Alsberg E. Buchman S. R. Kelly D. Laschke M. W. (2016). Tissue engineering and regenerative approaches to improving the healing of large bone defects. Eur. Cell. Mater 32, 87–110. 10.22203/ecm.v032a06 27434267

[B50] Wu X. Liu S. Chen K. Wang F. Feng C. Xu L. (2021). 3D printed chitosan-gelatine hydrogel coating on titanium alloy surface as biological fixation interface of artificial joint prosthesis. Int. J. Biol. Macromol. 182, 669–679. 10.1016/j.ijbiomac.2021.04.046 33857509

[B51] Wu X. Yan M. Lu J. Ge X. Li Y. Bian M. (2020). iRoot SP promotes osteo/odontogenesis of bone marrow mesenchymal stem cells via activation of NF-*κ*B and MAPK signaling pathways. Stem Cells Int. 2020, 6673467. 10.1155/2020/6673467 33424977 PMC7775135

[B52] Xiao L. Wu M. Yan F. Xie Y. Liu Z. Huang H. (2021a). A radial 3D polycaprolactone nanofiber scaffold modified by biomineralization and silk fibroin coating promote bone regeneration *in vivo* . Int. J. Biol. Macromol. 172, 19–29. 10.1016/j.ijbiomac.2021.01.036 33444651

[B53] Xiao Y. Xu M. Lv N. Cheng C. Huang P. Li J. (2021b). Dual stimuli-responsive metal-organic framework-based nanosystem for synergistic photothermal/pharmacological antibacterial therapy. Acta Biomater. 122, 291–305. 10.1016/j.actbio.2020.12.045 33359766

[B54] Xie C. Ye J. Liang R. Yao X. Wu X. Koh Y. (2021). Advanced strategies of biomimetic tissue-engineered grafts for bone regeneration. Adv. Healthc. Mater 10 (14), e2100408. 10.1002/adhm.202100408 33949147

[B55] Yadav L. R. Chandran S. V. Lavanya K. Selvamurugan N. (2021). Chitosan-based 3D-printed scaffolds for bone tissue engineering. Int. J. Biol. Macromol. 183, 1925–1938. 10.1016/j.ijbiomac.2021.05.215 34097956

[B56] Yang L. Chen S. Shang T. Zhao R. Yuan B. Zhu X. (2020a). Complexation of injectable biphasic calcium phosphate with phosphoserine-presenting dendrons with enhanced osteoregenerative properties. ACS Appl. Mater Interfaces 12 (34), 37873–37884. 10.1021/acsami.0c09004 32687309

[B57] Yang M. Lin L. Sha C. Li T. Zhao D. Wei H. (2020b). Bone marrow mesenchymal stem cell-derived exosomal miR-144-5p improves rat ovarian function after chemotherapy-induced ovarian failure by targeting PTEN. Lab. Invest. 100 (3), 342–352. 10.1038/s41374-019-0321-y 31537899

[B58] Yao Q. Cosme J. G. Xu T. Miszuk J. M. Picciani P. H. Fong H. (2017). Three dimensional electrospun PCL/PLA blend nanofibrous scaffolds with significantly improved stem cells osteogenic differentiation and cranial bone formation. Biomaterials 115, 115–127. 10.1016/j.biomaterials.2016.11.018 27886552 PMC5181114

[B59] Ye Y. Ke Y. Liu L. Xiao T. Yu J. (2021). CircRNA FAT1 regulates osteoblastic differentiation of periodontal ligament stem cells via miR-4781-3p/SMAD5 pathway. Stem Cells Int. 2021, 1–16. 10.1155/2021/5177488 PMC8731273 35003269

[B60] Yu L. Gao T. Li W. Yang J. Liu Y. Zhao Y. (2023). Carboxymethyl chitosan-alginate enhances bone repair effects of magnesium phosphate bone cement by activating the FAK-Wnt pathway. Bioact. Mater. 20, 598–609. 10.1016/j.bioactmat.2022.06.017 35846837 PMC9256840

[B61] Zheng L. Liu S. Cheng X. Qin Z. Lu Z. Zhang K. (2019). Intensified stiffness and photodynamic provocation in a collagen-based composite hydrogel drive chondrogenesis. Adv. Sci. (Weinh) 6 (16), 1900099. 10.1002/advs.201900099 31453055 PMC6702628

[B62] Zou Z. Wang L. Zhou Z. Sun Q. Liu D. Chen Y. (2021). Simultaneous incorporation of PTH(1-34) and nano-hydroxyapatite into Chitosan/Alginate Hydrogels for efficient bone regeneration. Bioact. Mater 6 (6), 1839–1851. 10.1016/j.bioactmat.2020.11.021 33336115 PMC7723774

