# Supplementary figures and images for: Phosphoserine-loaded chitosan membranes promote bone regeneration by activating endogenous stem cells
Source: Front Bioeng Biotechnol. 2023 Mar 23;11:1096532. doi: 10.3389/fbioe.2023.1096532 (PMC10076862; doi:10.3389/fbioe.2023.1096532)

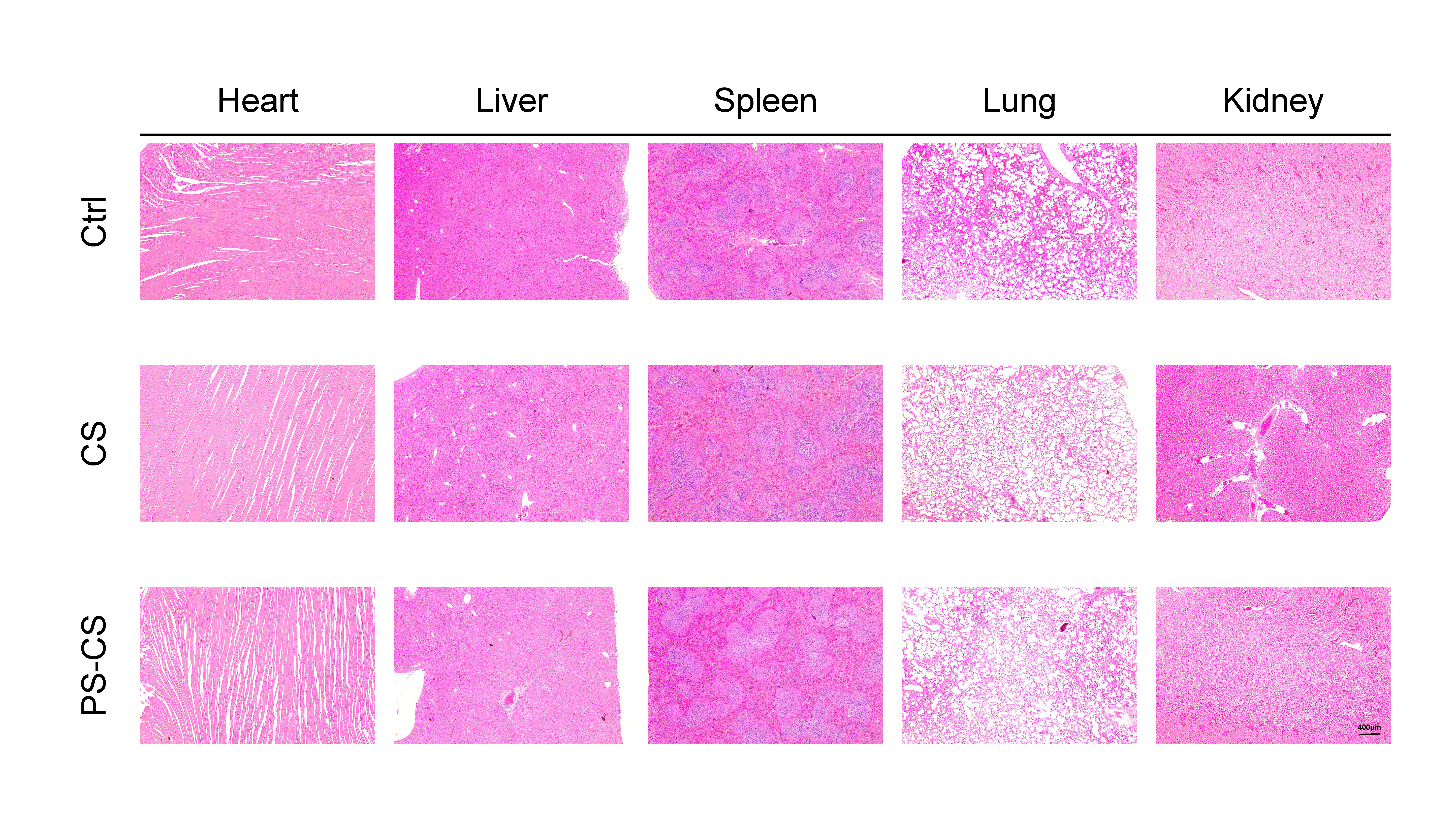

Supplement: Supplementary file 1 [file Image3.tif]

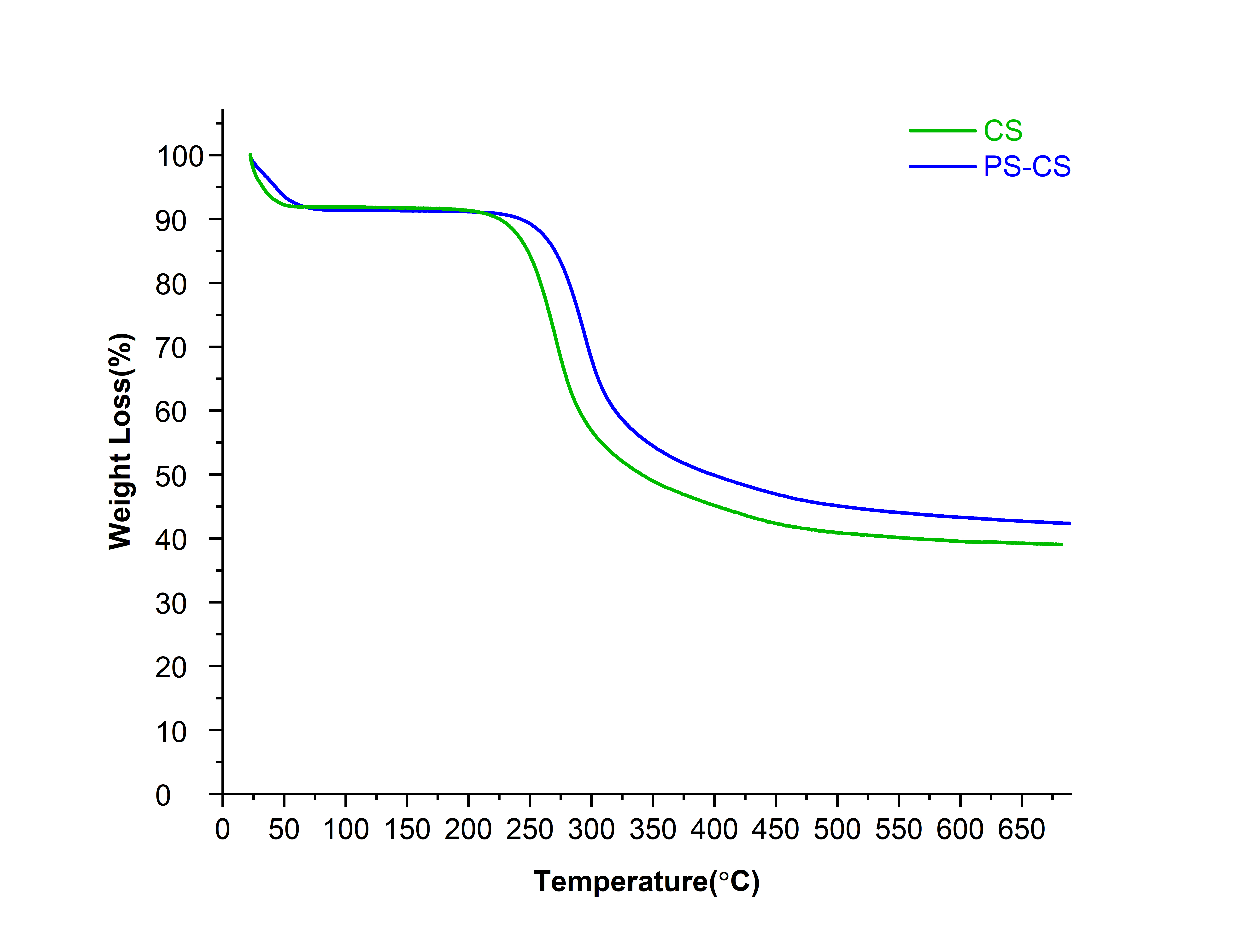

Supplement: Supplementary file 2 [file Image1.jpeg]

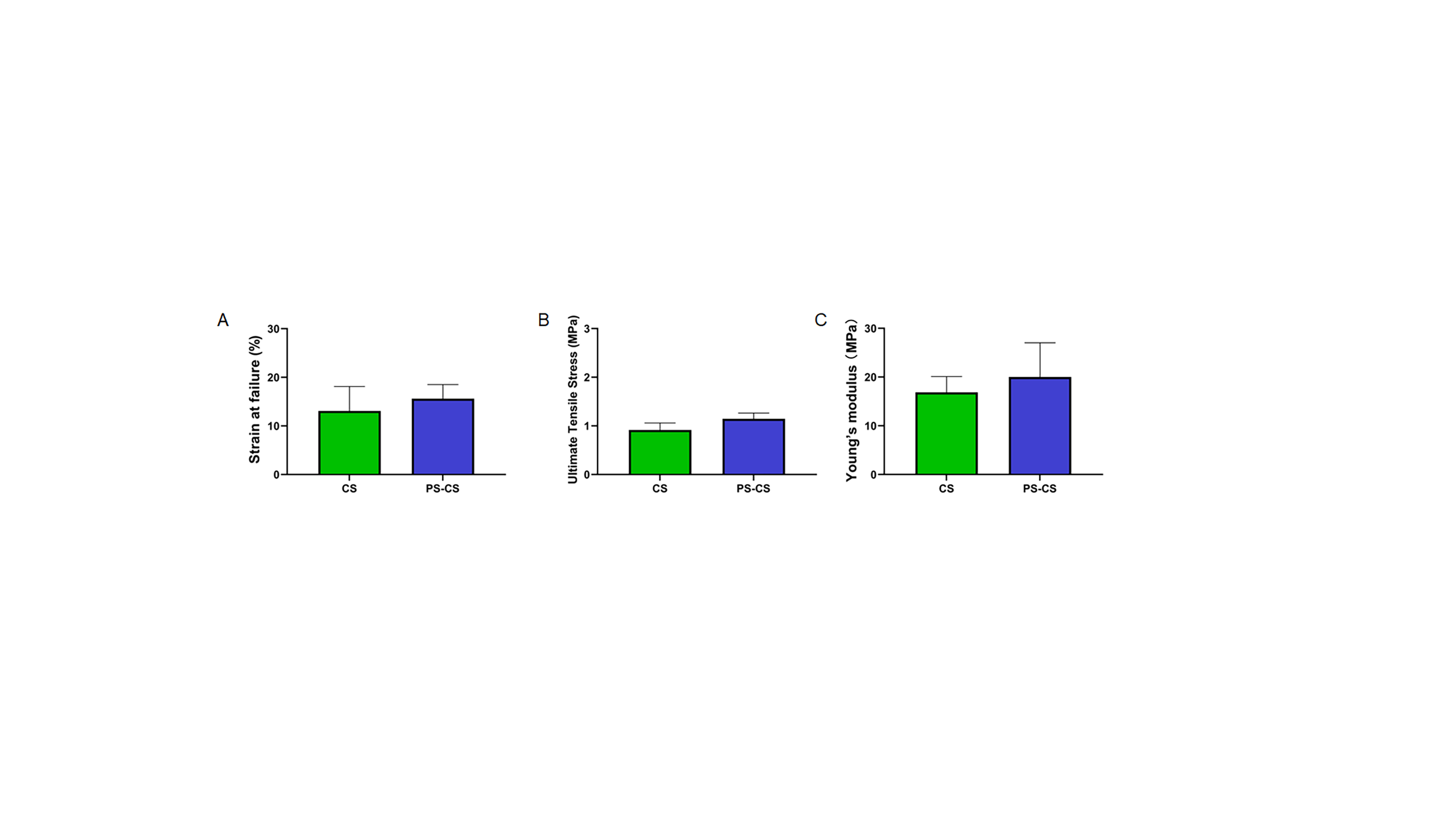

Supplement: Supplementary file 3 [file Image2.tif]
